# Observing temporal variation in hemolysis through photoacoustics with a low cost LASER diode based system

**DOI:** 10.1038/s41598-023-32839-3

**Published:** 2023-04-28

**Authors:** Soumyodeep Banerjee, Sandip Sarkar, Shaibal Saha, Sumit K. Hira, Subhajit Karmakar

**Affiliations:** 1 University Science Instrumentation Centre, The University of Burdwan, Bardhaman, 713104 India; 2 Applied Nuclear Physics Division, Saha Institute of Nuclear Physics, A CI of Homi Bhabha National Institute, Kolkata, 700064 India; 3 Department of Zoology, The University of Burdwan, Bardhaman, 713104 India

**Keywords:** Biophysics, Health care, Medical research, Engineering, Physics

## Abstract

Patients under hemolytic condition need continuous monitoring of lysis as depletion of Red Blood Cells (RBC) and the presence of antioxidant free hemoglobin (Hb) in excess amount due to hemolysis lead to severe deterioration of their health. Out of many modalities, Photoacoustics (PA) offers real time information noninvasively from deep lying blood vessels since Hb is the strongest chromophore in mammalian blood and the PA response of blood varies with the amount of Hb present. During hemolysis, total Hb content in blood however remains unchanged, thus, questions the use of PA in hemolysis detection. In this report, a hypothesis that the amplitude of the PA signal would not change with the amount of lysis is framed and tested by applying osmotic shock to the RBCs in hypotonic environment and the PA response is recorded over time using a low cost NIR based PA system. The experimental outcome indicates that PA amplitude falls off as lysis progresses in course of time consequently rejecting the hypothesis. The decaying PA response also carries the signature of RBC swelling during the early phase of lysis. The PA measurement can detect hemolysis as low as 1.7%. These findings further advocate transforming this NIR-PA system into a portable, noninvasive patient care device to monitor hemolysis in-vivo.

## Introduction

Erythrocytes or Red Blood Cells are one of the constituents of mammalian blood which transport oxygen to the cells of living tissues. Within each RBC, oxygen molecules bind to a protein called hemoglobin. In many hematological and non-hematological diseases like spherocytosis, thalassemia, hemolytic anemia, malaria etc., or in snake bite where venom is injected, or the application of hemotoxic drug or drug carrying nanoparticle for treatment purpose or in some clinical procedures, surgical intervention or in injuries due to accidents, RBCs get damaged or destroyed and the packed Hb molecules are released into the plasma by a process called hemolysis^1–8^. The patients with hemolysis need immediate clinical attention as the oxygen delivery to the cells gets seriously hampered due to the loss of RBCs. Even the presence of extracellular Hb in excess amount also creates oxidative damage to the vasculature and exposed tissues^9^. Continuous monitoring of hemolysis either in-vivo or in-vitro is thus a subject of patient interest.

Once the Hb is released upon rupturing of the cell membrane, the golden yellowish blood plasma turns reddish and the amount of light that is absorbed continues to vary with the amount of free Hb. The optical absorption of cell-free plasma has been utilized in earlier studies to find any trace of Hb and quantify the degree of hemolysis either by visual inspection of the change in plasma colour or comparing different levels of absorptions at multiple wavelengths^10–13^. Optical spectrophotometry based in-vitro studies produce accurate results, however, face technical challenges in noninvasive implementation.

In most of the experiments, hemolysis is simulated by imparting an osmotic shock to the RBCs with the help of hypotonic media. The time dependent variation in osmotic pressure induced lysis passes through different transformational stages like RBC swelling, hole formation and release of Hb into plasma. Observing these stages can be of immense use to study the health of a RBC^14^. The optical techniques discussed above are solely concerned with the presence of free Hb, hence cannot offer adequate information about these transformations taking place in a lysing RBC suspension^15^.

A temporal variation in optical density in hemolysing samples was reported earlier^1^. The in-vitro study did not account for only the free Hb, also examined the change in the whole cellular suspension. But any experimental evidence of the different stages the RBCs are going through is absent. A chemical nanofilter based hemolysis detection is presented by Zhou et. al. where the filter separates whole RBCs from plasma and then examines the plasma using an optofluidic sensor that measures evanescent absorption^16^. This study avoids extensive sample preparation but is in-vitro and complex. Different transformational phases of the RBCs are also untraceable while using this technique to study hemolysis. Electrical experiments like measurement of capacitance and conductance to study hemolysis as well as RBC membrane fragility have been reported^15^. Dynamic information of the membrane and the cytoplasm of the RBCs can be studied by this experiment very well. A simple and real time hemolysis monitoring setup has recently been published which measures the change in capacitance of the blood due to the dispersion of Hb into blood plasma^17^. The electrical systems to detect real time hemolysis though appear simple in design; they are not in-vivo.

Photoacoustics (PA) is the study of ultrasound signals emitted by a sample due to thermoelastic expansion while absorbing pulsed or temporally varying optical energy (of the order of ns, typically). Since the acoustic scattering inside biological tissue is few orders of magnitude less than the optical scattering, PA technique can harvest information from greater depth (>> 1 mm) compared to the optical techniques^18^. Not only that, this hybrid modality offers excellent signal contrast based upon the optical contrast present within the sample thus emerges as a promising noninvasive biomedical imaging technique to view organs to organelles in-vivo.

The conventional PA systems can be designed as noninvasive probes to provide real time information during clinical procedure but they cannot be made compact and cost-effective because of the bulky and expensive optical sources like Q-switched Nd:YAG LASERs and dye LASERs. Patient care devices, on the other hand, are desired to be portable and cost effective too. In recent times, a small footprint approach has been taken up by several researchers employing low power and low cost diode based optical sources like pulsed LASER diode (PLD), continuous LASER diode or high power LED. LASER diode based imaging systems have been used to image different in-vitro phantoms like thread embedded chicken breast as well as in-vivo samples like mouse ear^19,20^. Photoacoustic quantification experiments on total hemoglobin from human radial artery and blood glucose from human forefinger have also been performed^21–23^. High power LEDs have also been incorporated as optical sources in photoacoustic imaging and tomography including different biomedical applications^24,25^. Some experiments regarding frequency domain photoacoustic microscopy were performed using high power LEDs as the source of illumination^26,27^. A recent work on photoacoustic quantification by spectral unmixing technique was also reported to be using high power LEDs^28^. In addition to these, various other photoacoustic systems with low cost optical sources and their applications can be found in the review article by Singh, M. K. A. & Xia, W.^29^.

Hb molecules, absorbing the irradiation in the UV–Vis-NIR region, enable PA technique to probe any changes in the ensemble of RBCs and its environment. Compared to the UV-Vis region, Hb molecule has a moderate absorption coefficient peaking around 970 nm for oxy-Hb and 910 nm for deoxy-Hb as well as a very low scattering coefficient^30^.Thus, NIR wavelengths, lying inside the optical window of human skin, makes itself a suitable choice for extracting information especially from deep lying blood vessels. Previously, photoacoustic estimation of Hb in-vivo and in-vitro has been performed^31,32^. The variation in the PA signal with varying hematocrit has also been studied^33,34^. Some recent studies further reveal that the frequency response of the PA signal carries the signature of the shape of the RBC^35^.

In this report, an in-vitro experimentation has been presented to observe temporal variation in hemolysis through photoacoustics. As the total Hb content in blood remains unchanged throughout the hemolysis process, the proposed experiment tests the hypothesis that the amplitude of the photoacoustic waveform would not change with the temporally varying amount of lysis, irrespective of the osmolarity of the environment. Hemolysis is performed by leaving RBCs into different strengths (Osmolarity: 134.75 mM, 125.125 mM, 115.5 mM, 96.25 mM, 77 mM and 38.5 mM) of the hypotonic saline environment. To test the hypothesis, a table top and cost effective PA system has been designed and fabricated that consists of a 905 nm pulsed LASER diode as the illumination source. If this in-vitro study detects significant changes in the PA response with respect to the amount of lysis, the use of NIR excitation, envisaging on the findings of earlier researchers^22,23,36,37^, would enable the system to be transformed into a noninvasive patient care device to monitor hemolysis in-vivo.

## Materials and methods

### Packed cell volume preparation

Packed cell volume (PCV) is the total cellular volume of a blood sample, which is mostly contributed by RBCs (> 99%). To extract PCV, porcine whole blood was obtained from a slaughterhouse and immediately mixed with Ethylene Diamine Tetra Acetic Acid (EDTA) at 3 g/L. Then the blood was washed to get rid of plasma and all other debris. The washing process is as follows:

(i) At first the raw whole blood was distributed into centrifuge tubes and centrifuged at 4000 rpm (~ 700*g*) for 10 min to separate the non-cellular and cellular elements as supernatant and precipitate.
(ii) The cell-free supernatant was collected very carefully from the top.
(iii) Then commercially available normal saline was mixed with the leftover precipitate, which is actually concentrated RBC.
(iv) Then the sample was centrifuged again at 4000 rpm for 10 min.
(v) All the actions from step (ii) to step (iv) were repeated thrice to get a clear colourless supernatant.

At last, the precipitate, which is the PCV, was collected by separating the colourless supernatant from the top.

### Lysing media preparation

Commercially available normal saline (0.9% or 154 mM of NaCl in water), which is isotonic to RBC is mixed with distilled water in 7:1, 13.3, 3:1, 5:3, 1:1 and 1:3 (v/v) ratios. The resulting hypotonic solutions were of 134.75 mM, 125.125 mM, 115.5 mM, 96.25 mM, 77 mM and 38.5 mM NaCl in water respectively.

### Lysing, non-lysed and totally lysed sample preparation

Packed RBC and lysing solutions were mixed in 1:4 (v/v) ratio, resulting into a suspension of 20% PCV. The lysing samples were prepared in adequate amounts to meet the requirements of both the optical absorption and PA experiments. This was done to maintain consistency in any possible error generated in the sample preparation process.

Separately two 20% PCV samples were prepared, one in normal saline (non-lysed) and the other in ammonium chloride solution (totally lysed).

### Photoacoustic system development

**Figure 1:**
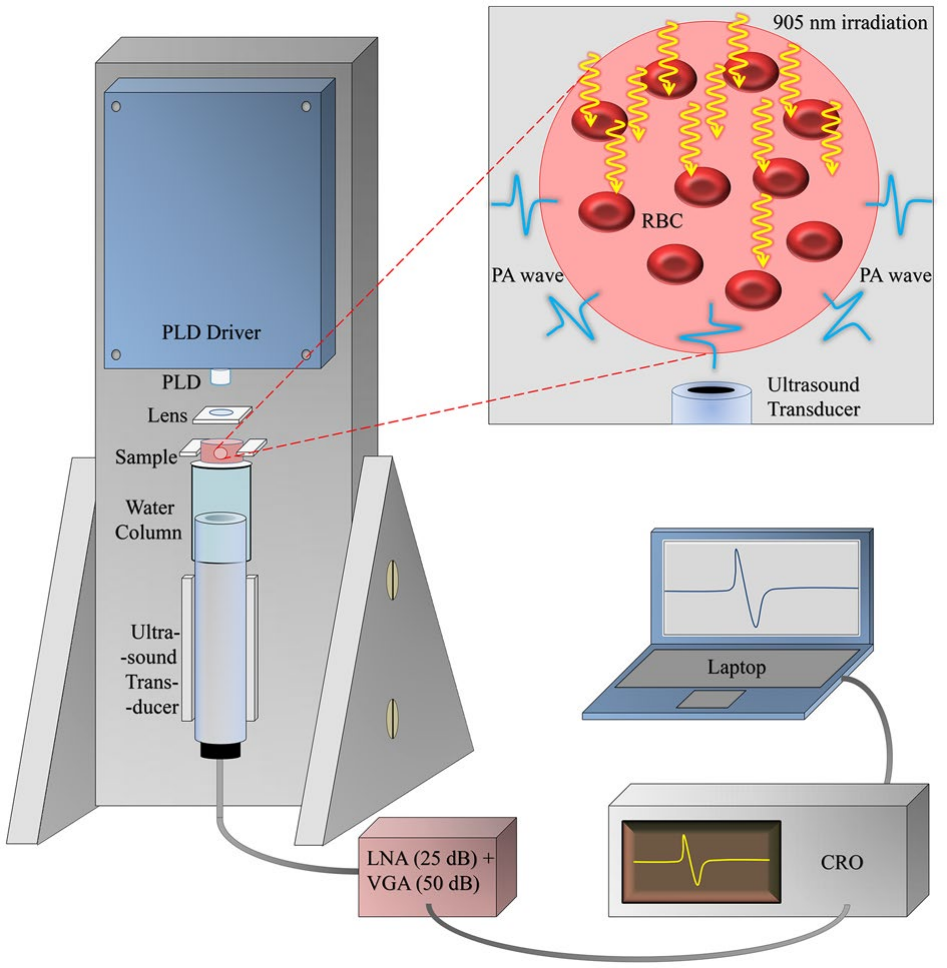
A schematic of the low cost, small footprint photoacoustic system consisting of a 905 nm pulsed LASER diode, a 10 MHz ultrasound transducer placed on a Perspex body in inverted microscope manner. The PA signal received by the transducer is fed to the digital oscilloscope through a cascade of one 25 dB LNA and one 50 dB VGA. Digitized PA waveform is transferred to a laptop computer using Python code and processed. The inset is showing an ensemble of RBCs which absorb pulsed NIR excitation and emit omnidirectional PA wave by thermoelastic expansion.

A small-footprint PA system was developed using a 75 W, 905 nm pulsed LASER diode (SPL PL90_3, OSRAM) and an unfocused ultrasound transducer of centre frequency 10 MHz (Panametrics, V312). A custom made microcontroller based pulse generator was used to trigger the LASER diode driver (PCO 7121, Directed Energy Inc.). For this experiment, trigger pulses of width 110 ns and repetition frequency of 500 Hz were generated. The high voltage to the LASER diode driver was set to 35 V. The laser source along with the driver and the transducer were fitted onto a Perspex structure in an inverted microscope fashion (Fig. 1). The sample holder is placed in a manner where the sample container can sit on the top of the sensing face of the ultrasound transducer, separated by a water column of 6 mm height. A collimator lens (EFL 6 mm) is placed in between the PLD and the sample surface. The output signal of the ultrasound transducer was amplified through a cascade of a low noise amplifier (25 dB, ZFL 500, Mini circuits) and a variable gain amplifier (50 dB, Picopulser, US Ultratek). The amplified output was fed to a low cost digital oscilloscope (Tektronix 1102B).

### Photoacoustic experimentation and data acquisition

Each sample (including non-lysed and lysing samples) was poured once at a time into a sample container according to its capacity and the container was put into the system to record the PA response. This process was repeated at every time point of data acquisition. The data acquisition was done using two different modalities. All the data after the first 3 min of experimentation were pulled using a laptop computer by a Python code via usb communication with the CRO. The data within the first 3 min time were acquired in a different way. The conventional data acquisition by laptop computer was a process of around 10 s in this case. As the experiment was to capture the dynamic variation in the responses, so that a 10 s time period to capture data was not affordable where the responses were changing in a very fast manner. To capture the waveform of those time points, the display of the CRO was photographed using a smartphone camera and then the datasets were extracted using an image processing technique performed on the captured images in a laptop computer. The datasets in this case were sampled into an equal number of data points with computationally acquired datasets. As the curves displayed on the CRO screen were averaged over 128 datasets and the datasets computationally acquired from the CRO were also averaged over 128 datasets, so finally all the datasets were equivalent by that mean. The PA amplitude of 20% PCV in normal saline is considered as the data point of t = 0 min for every medium and is further used for normalization and plotting.

### Optical absorption measurement and lysis estimation

The optical absorptions of the non-cellular liquid part of the samples were to be measured to find the Hb content at those moments. The sample containing ammonium chloride media was kept for 4 h and then centrifuged at 12,500 rpm for 10 min and the supernatant was collected. Similarly, the sample of RBC in normal saline is centrifuged at 4000 rpm for 10 min and the supernatant was collected. These were designated as the 100% lysed and 0% lysed supernatants respectively.

The supernatants of the lysing samples were to be collected after centrifuging at 4000 rpm for 10 min. Before centrifugation, lysis processes in all the samples were to be stopped in order to restrict Hb release by the lysing RBCs anymore. So, at the time points of data acquisition, all the lysing samples were mixed into normal saline in 1:10 (v/v) ratio. This treatment effectively arrested the lysis process. For consistency, the supernatants of 0% lysed and the 100% lysed samples were also mixed with normal saline in the same ratio. After that, all these samples were centrifuged at 4000 rpm for 10 min and the supernatants were collected for optical absorbance measurement. Optical absorbance values of all the supernatants were measured in a 96-well plate reader (Multiskan GO, Thermo Fisher Scientific).

After acquiring the absorbance data, at first the values were corrected by subtracting the absorbance of the 0% lysis sample (A_0_) from all the other absorbance values. Then the corrected values were normalized by the corrected absorbance value of the 100% lysed sample (A_100_). The resulting values were multiplied by 100 to get the lysis percentages $\left(\frac{A-{A}_{0}}{{A}_{100}-{A}_{0}}\right)$.

## Results and discussions

**Figure 2:**
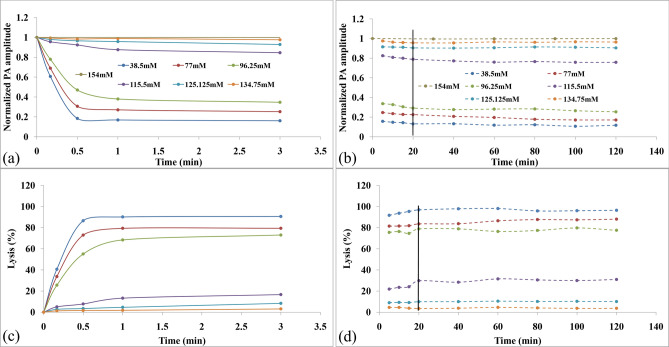
(**a**) Variation in normalized PA amplitude with time for the first 3 min of experimentation, showcasing the rapid phase of biphasic PA amplitude variation roughly till 0.5 min, (**b**) the same for 3 min to 120 min, showing the steady state i.e. the plateau, (**c**) variation in lysis percentage with time for the first 3 min, showing rapid phase within the first 0.5 min, (**d**) plateau of lysis percentage variation with time from 3 to 120 min.

Hemolysis of packed cells in six hypotonic saline media of strengths 134.75 mM, 125.125 mM, 115.5 mM, 96.25 mM, 77 mM and 38.5 mM has been investigated at different time points (0.167 min, 0.5 min, 1 min, 3 min, 5 min, 10 min, 15 min, 20 min, 40 min, 60 min, 80 min, 100 min and 120 min) with respect to the PA response. The percentage of lysis is also determined for the same samples at the same instances by measuring optical absorption of the supernatant. As the total Hb gets redistributed between the RBCs and the medium, at each instance of lysis, the PA responses can be assumed as additive resultants of the contribution of whole RBCs left in the sample and the same from free Hb molecules dispersed in the medium. The assumption that total Hb remains unchanged leads to the hypothesis: the PA response would not change significantly with time. Contrary to the hypothesis proposed, it is found that the normalized PA amplitude does not remain constant over time rather decays irrespective of the tonicity of the medium (Fig 2a). The decaying trend of PA response appears to be correlated with the increase of free hemoglobin over time (Fig. 2b). For example, when the release of Hb from the RBCs in the environment is least (1.25% and 2.75% lysis within the first 0.5 min) that is in the 134.75 mM and 125.125 mM media, the PA response displays the slowest fall off (0.67% and 2.1% within the first 0.5 min). A significantly greater fall off (69.34% and 81.57% within the first 0.5 min) in PA amplitude is observed in the environment of 77 mM and 38.5 mM in which the release of Hb is much higher (72.94% and 86.53% lysis within the first 0.5 min) due to greater osmotic shock to the RBCs. The other hypotonic media, 115.5 mM and 96.25 mM, impart comparatively moderate osmotic shock to the RBCs and cause a moderate amount of lysis (in between 7.8% to 55.09%) which corresponds to 7.6% to 52.97% reduction in PA amplitude within the first 0.5 min

The faster rate of decay of PA amplitude $\left(\frac{\Delta PA}{\Delta t}=\frac{PA\left({t}_{2}\right)-PA\left({t}_{1}\right)}{{t}_{2}-{t}_{1}}\right)$ (Table 1) for the RBCs in the environment of 96.25 mM, 77 mM and 38.5 mM, does not continue beyond 30 s (Fig. 2b). This phase can be designated as the Rapid Phase, for the ease of discussion. The rate of decay slows down after 3 min and the PA response reaches a plateau at the time point of 20 min (Table 1). During the same period of time the hemolysis also becomes almost steady (Fig. 2d) resulting into negligibly small values of rate of percentage of lysis $\left(\frac{\Delta L}{\Delta t}=\frac{L\left({t}_{2}\right)-L\left({t}_{1}\right)}{{t}_{2}-{t}_{1}}\right)$, thus establishes a correlation with PA amplitude. Temporally biphasic nature of the hemolysis process in hypotonic environment, previously experienced by other researchers in the optical and electrical studies^1,15^, is being reflected in the PA response too. To appreciate the amplitude fall off due to lysis in different hypotonic environments, the PA responses of the packed cells (PCV = 20%) in normal saline (i.e., in the isotonic environment) with respect to time are also depicted in Fig. 2a. The data points show almost no variation with time. As the cells remain in the isotonic environment no osmotic pressure gradient is developed between outside and inside of the cell membrane to initiate lysis.

**Table 1:** Rate of change in PA amplitude and lysis percentage.

Osmolarity (mM)	Rapid phase (t_2_ = 0.5 min, t_1_ = 0.167 min)		Plateau (t_2_ = 120 min, t_1_ = 20 min)	
	$\frac{\Delta PA}{\Delta t}$	$\frac{\Delta L}{\Delta t}$	$\frac{\Delta PA}{\Delta t}$	$\frac{\Delta L}{\Delta t}$
134.75	−0.03107	1.31	−0.00015	0.00
125.125	−0.04210	2.25	−0.00055	0.00
115.5	−0.09613	8.24	−0.00038	0.01
96.25	−0.92875	88.69	−0.00031	−0.01
77	−1.14980	117.66	0.00000	0.04
38.5	−1.26760	137.76	−0.00007	0.00

In hypotonic solutions, RBCs start absorbing water from the environment to become isotonic with respect to it. As a result, RBCs swell and some of them burst. Upon destruction of those RBCs, the cytoplasmic content gets mixed with the environment and increases the medium tonicity. On the other hand, the RBCs which still remain unlysed, absorb water from the environment to reduce the tonicity of their cytoplasm. This reduction in tonicity difference between RBC cytoplasm and environment tends to decrease swelling and lysis rate by reducing the osmotic pressure. With increasing swelling, the water permeability of the RBC membrane falls off. This internal inhibitory feedback restricts further RBC inflation. In the rapid decay phase (Fig. 2a,c), when the spatial gradient of tonicity inside and outside the RBC membrane is high, the major part of the lysis happens. With the increase in the amount of lysis, the tonicity difference between inside and outside of the RBC membrane decreases. At some point, it fails to exert enough osmotic pressure to overcome the membrane resistance toward water influx, thus to rupture the membrane of most of the leftover inflated RBCs. This dynamic process indicates the transition between the Rapid Phase and the plateau. In this later stage i.e. in the plateau region (t > 20 min), the lysis process almost stops.

**Figure 3:**
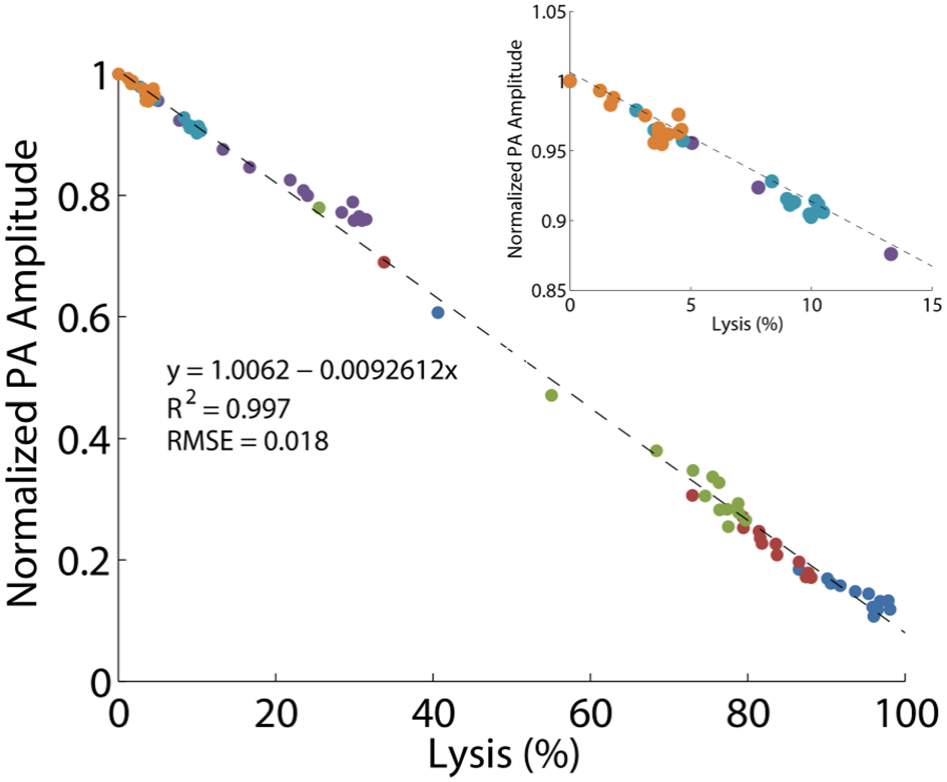
A crossplot of lysis percentage and normalized PA amplitude of the samples of all six osmolarities. Same colour code is followed as in Fig. 2. All the datapoints are fitted using a common trendline, which is a straight line in nature. It indicates that, independent of the tonicity of the environment, the variation in PA amplitude with lysis would follow a single straight line. This line can be used as the calibration curve to estimate the percentage of lysis by using the PA amplitude of the sample acquired using the present setup. In set, an enlarged version is presented within the lysis level of physiological interest.

The correspondence observed (Fig. 2) between the amount of lysis and the PA response primarily suggests a functional relationship between the two. In Fig. 3, a crossplot between normalized PA amplitude and lysis percentage depicts that datapoints, corresponding to the samples in the environment of six osmolarities, are well distributed on and with respect to a straight line. A high value of Goodness of Fit (R^2^ = 0.997) and a small error of regression (RMSE = 0.018) indicate that independent of the tonicity of the environment, this regression line can be used as a calibration curve to estimate the percentage of lysis from the PA amplitude of the sample acquired using the present setup. The regression line with a non-zero slope further rejects the hypothesis that the amplitude of the PA wave would not change with respect to the amount of lysis (p-value for the test is << 0.001).

The data points near to the vertical axis of the plot (orange circles of inset Fig. 3) can be used to decide the detection threshold of the hemolysis. The nearest one that corresponds to 0.7% change in PA amplitude reflects a 1.25% lysis, minimum amount estimated by this experiment. However, that change in PA amplitude falls in the fringe zone of the voltage resolution of the oscilloscope (0.6% change corresponding to 0.195 mV for this measurement). If a change of 1% in the PA amplitude is considered as a threshold, it will correspond to the next data point and indicate almost 1.7% lysis of RBCs (inset Fig.3).

The linear dependence between the PA response and the amount of lysis can be rationalized with the help of the following observations: (i) confinement of Hb molecule enables each RBC to act as a cellular PA generator^38,39^, (ii) since the scattering coefficient of RBC suspension is small in the NIR range, the number of internal reflections of photons inside the optically dense RBC increases the probability of absorption of a photon by the RBC. As a result, RBCs upon NIR excitation generate a significantly higher amplitude of PA signal than the same amount of encapsulated Hb while mixed in the same volume of plasma or any similar isotonic buffer^34^ and (iii) the PA amplitude varies almost linearly with the number density of RBC in the hematocrit range from 0 to 20%^34,40^.The amount of hemolysis is directly proportional to the number of RBCs destroyed i.e., the fall in the number density of PA sources which in turn decreases the amplitude of the PA signal linearly according to observation (iii).

Swelling of RBCs due to net inflow of water decreases the refractive index (rI) of the cytoplasm^41^. On the other hand, the release of Hb molecules due to membrane rupture increases the same of the surroundings^42^. During lysis, these two processes reduce the difference between refractive indices of inside and outside of the erythrocyte cell wall. This is why the inflated RBCs have very low optical contrast with respect to the surroundings and are hard to be recognized via optical microscopes, often referred as “Ghost RBCs”. The difference of refractive indices as decreases, the number of internal reflections of photons inside the RBC falls off, consequently reducing the optical energy absorption by the RBC^43^. Because of the drop in optical absorption, the inflated RBCs fail to generate PA signal as intense as the normal RBCs. Nevertheless, the contribution of the inflated RBCs to the PA response becomes appreciable when the same coming out of extracellular Hb is little or negligible i.e., when the amount of lysis of RBCs is very small as well as the inflated volume is retained for a significant period of time. It happens to the RBCs suspended at less hypotonic (134.75 mM and 125.125 mM) media and experienced mild osmotic shock at the early phase of lysis. The PA response of the inflating RBC is likely to be different at different moments as the rate of change of shape of a RBC does not remain constant over the time period^15^. It is therefore expected to observe a localized nonlinear dependence of PA amplitude on the amount of lysis at the earliest time points.

**Figure 4:**
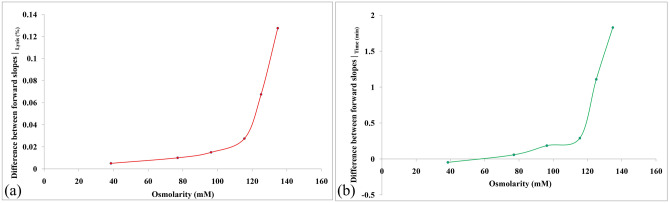
Difference between two forward slopes with (**a**) lysis percentage and (**b**) time as parameters are showing high values for higher osmolarities like 134.75 mM and 125.125 mM, which indicates nonlinear dependency between lysis percentage and PA amplitude at those data points, implying the presence of inflated erythrocytes in those samples.

Difference between forward slopes ${\left(\frac{\Delta PA}{\Delta L}\right)}_{{L}_{2}\left({t}_{2}\right)}-{\left(\frac{\Delta PA}{\Delta L}\right)}_{{L}_{1}\left({t}_{1}\right)}$ at two lysis percentages (L_2_, L_1_) as well as two time points (t_2_, t_1_) are estimated and plotted against osmolarity of the lysing solution in Fig. 4. It is found in either cases that the difference between slopes are neither zero nor negligibly small for the solutions of higher osmolarity. The values rather appear small for the solutions of lower osmolarity and grow with the increasing osmolarity. It concludes that, at those lysis values (0.6%, 0.9% and 1.2%) as well as time points (0 min, 0.167 min and 0.5 min), the relation between PA amplitude and lysis is predominantly nonlinear for the solutions of higher osmolarity (134.75 mM and 125.125 mM), whereas for other lysing media (115.5 mM, 96.25 mM, 77 mM and 38.5 mM), it is almost linear. The nonlinear nature indicates the effect of swelling in the PA response. This is found to be in good agreement with the observations of RBC swelling by A. Pribush et al.^15^ through conductivity and capacitive measurement on lysing blood. In the other four hypotonic environments, the absence of inflated RBCs is prominent. Due to high osmotic shock, almost instantly the RBCs get destroyed, so mostly the normal or little inflated RBCs are left^15^ to contribute to the PA signal. It makes the PA amplitude to vary linearly with lysis percentage. It is also to be noted that, as the percentages of lysis considered for the estimation of slopes are very small, the local nonlinearity between PA amplitude and amount of lysis does not affect the global linear trend of the same depicted in Fig. 3.

Another local nonlinearity can be observed near the extreme lysis end of the data points of Fig. 3. The possibility of it originating either from the contribution of Hb or RBC separately can be ruled out as the variation of normalised PA amplitude with respect to increasing Hb density equivalent to PCV 0% to 20% as well as with respect to increasing PCV 0% to 20% are highly linear (Fig. S1). It might have been generated by the complex optical absorption phenomena happening in the microscopic scale. At the highly lysed end, there are very few inflated RBCs left in the free Hb enriched media. The changes in refractive index of both the sides of RBC wall, the change in scattering coefficient due to the change in shape of the RBCs etc. affect the optical absorption process and makes it a very complicated one. How does this complexity in optical absorption actually contribute to the nonlinearity at the highly lysed end of the respective figure, can be a topic of a separate study.

**Figure 5:**
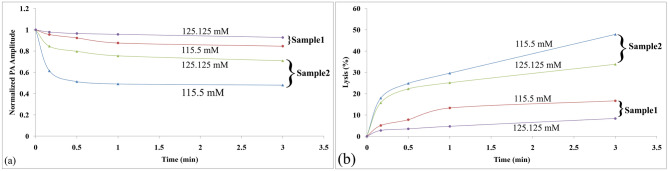
Result of similar measurement done on a different porcine blood sample (sample 2) in 115.5 mM and 125.125 mM osmolarity, showcasing different rate of change of (**a**) PA amplitude as well as (**b**) lysis percentage with time (min) indicating higher cell fragility compared to the primarily reported sample (sample 1).

A similar PA measurement has been performed on a different blood (porcine) sample with the same amount of packed cells (PCV = 20%) in the 115.5 mM and 125.125 mM saline environment respectively. As most of the lysis occurs within the first three minutes, the temporal variation in PA amplitude for this new sample i.e. Sample 2 is compared to that of Sample 1 within the same interval of time. A decaying trend of PA amplitude over time is also observed for the new sample (Fig. 5a). However, in comparison with Sample 1, a rapid fall off in the PA amplitude is noticed irrespective of the environment (−0.3037 in 115.5 mM and −0.14383 in 125.125 mM). The faster rate of decay in the PA amplitude for the new one is substantiated by more release of Hb i.e. greater amount of lysis in the same environment (20.76 in 115.5 mM and 19.73 in 125.125 mM) (Fig. 5b). The amount of lysis happened to the new sample in either environment is found to be more than double of the same for Sample 1 at every instant (Fig. 5b) in a period of 3 min. This observation suggests that the RBCs of the new sample are more fragile than the previous one and concludes that the falling rate of PA amplitude increases with the increase in fragility of the sample. This result opens up another scope of the present setup to be involved in the RBC fragility test.

Each RBC while absorbing brief optical radiation emits an N-shaped acoustic pulse by thermoelastic expansion. Weighted sum of these N-shaped wavelets with different relative path delays determines the shape (frequency) and strength (amplitude) of the PA signal for an ensemble of cells, at the receiver. When the number density of RBCs falls off during hemolysis, the number of wavelets contributing in-phase or almost in-phase to the weighted sum also falls off. As a result the amplitude of the PA signal drops and simultaneously the frequency content gets changed, likely to be shifted towards the frequency of individual wavelets (Figs. S2–S5). Therefore, PA amplitude and frequency both are functions of the number density of RBCs and their spatial distribution. This description is valid for the ensemble of cells. However, with the depletion of cells, the density of free Hb in the surrounding fluid grows and after a certain amount the PA contribution of the surroundings, which has a lesser optical absorption^34^ and different geometry, becomes significant. The PA emission of this bulk source is expected to have reduced amplitude and frequency than the response of the ensemble of cells. The PA signal of the mixture of ensemble of RBCs and free Hb enriched homogeneous solution thus can be described as a linear sum of two signals having different amplitudes as well as frequencies. Since cellular structure offers enhanced optical absorption the amplitude of the resultant signal is dictated by the amplitude of the ensemble response till degree of hemolysis is not too high (linear relationship with high correlation in Fig. 3). Near completion of hemolysis, a few cells are left to ignore their in-phase or almost in-phase addition in PA response which results in a drop in amplitude but increase in frequency (bandwidth and centre frequency) (Fig. S6). No doubt, this can be a subject of future research to observe temporal variation in hemolysis through photoacoustics when the sample is sufficiently sparse (even though the situation is not clinically significant) to ignore in-phase or almost in-phase addition. Apart from the number density of RBCs, other factors like receiver parameters (bandwidth and aperture), optical beam parameters (beam width, pulse width, energy) may be playing significant roles in visualizing the amplitude fall off. At any clinically significant hematocrit level, the number density of cells is not at all small to ignore the in-phase and almost in-phase addition of their PA responses, therefore it can be argued that the trend of fall in PA amplitude with hemolysis, as observed in this experiment, is not likely to change with the receiver parameters as well as optical beam parameters. Nevertheless, the design of a low cost PA system can be further optimized with respect to them and the associated electronics.

Photoacoustic amplitude of a blood sample strongly depends on the Hb concentration, hematocrit level and oxygen saturation of each Hb molecule. The effect of oxygen saturation on the PA amplitude can be minimized by selecting the excitation wavelength close to the isosbestic point of the Hb absorption spectra. Hb concentration as well as hematocrit level varies from person to person depending upon the factors like race, gender, age and health condition. These factors may be considered as additional input to the calibration curve presented in Fig. 3 in order to estimate hemolysis quantitatively while translating the present technique to a patient care device. Furthermore, the in-vivo quantitative estimation needs local optical fluence compensation as the optical fluence to the same targeted vessel varies due to variation in optical properties of biological tissues from one patient to another. The realistic numerical Monte Carlo simulations and an analytical approach based on the beam-spread function^44^ have been used to model light transport in highly scattering media like biological tissues (e.g. brain tissue). An iterative algorithm has been also proposed to recover absorption coefficients from optical absorbed energy maps based on a 3D Monte Carlo simulation of light transport^45^. Similar approaches can be adopted in future study of the PA technique to estimate hemolysis in-vivo. Nevertheless, the in-vitro PA technique presented here with a little modification to the source-detector geometry can be incorporated to clinical practice for detecting the real time changes in hemolysis qualitatively.

## Conclusion

It is observed from the present study that the amplitude of the PA signal does not remain constant throughout the time course of lysis though total Hb remains constant. Instead it decays with increasing amount of lysis over time. Decaying the photoacoustic response manifests the temporally biphasic nature of hemolysis and also reflects the presence of the inflated state of RBCs at the early phase of lysis. Different decay rates of PA amplitude for different samples measured under identical experimental conditions potentialize this PA system to test the fragility of RBC membranes or the health of the RBCs. The in-vitro PA measurement is also able to detect hemolysis as low as 1.7%. The above findings further suggest the transformation of this NIR-PA system into a portable, noninvasive patient care device for monitoring hemolysis in-vivo. It may be noted that PA responses corresponding to different degrees of lysis are degenerate as they refer to the same amount of Hb. Therefore, any patient care device (ref. to “Introduction”) that measures PA responses from the blood cannot estimate Hb density reliably unless the lysis condition (degree of lysis, swelling status of RBCs etc.) is known a priori. This is a very important statement, can safely be made by the findings of this study, having a large impact on the scope of developing PA device for hematological diagnosis.

## Supplementary Information

Supplementary Information.

## Data Availability

The data that support the findings of this study are available upon reasonable request to the corresponding author Subhajit Karmakar.

## Abbreviations

PA: Photoacoustics
RBC: Red blood cell
Hb: Hemoglobin
PCV: Packed cell volume
NIR: Near infra-red
