# Supplementary material for: Observing temporal variation in hemolysis through photoacoustics with a low cost LASER diode based system
Source: Sci Rep. 2023 Apr 28;13:7002. doi: 10.1038/s41598-023-32839-3 (PMC10147907; doi:10.1038/s41598-023-32839-3)
Supplement: Supplementary file 1 — Supplementary Information. [file 41598_2023_32839_MOESM1_ESM.pdf]

Observing Temporal Variation in Hemolysis through Photoacoustics with a Low Cost LASER Diode Based System

Soumyodeep Banerjee[1], Sandip Sarkar[2, *], Shaibal Saha[2], Sumit K. Hira[3], Subhajit Karmakar[1]

[1] University Science Instrumentation Centre, The University of Burdwan, Bardhaman, India

[2] Applied Nuclear Physics Division, Saha Institute of Nuclear Physics, Kolkata, India

[3] Department of Zoology, The University of Burdwan, Bardhaman, India

* Retired Professor

Contact: skarmakar@usic.buruniv.ac.in

Supplementary Figure:

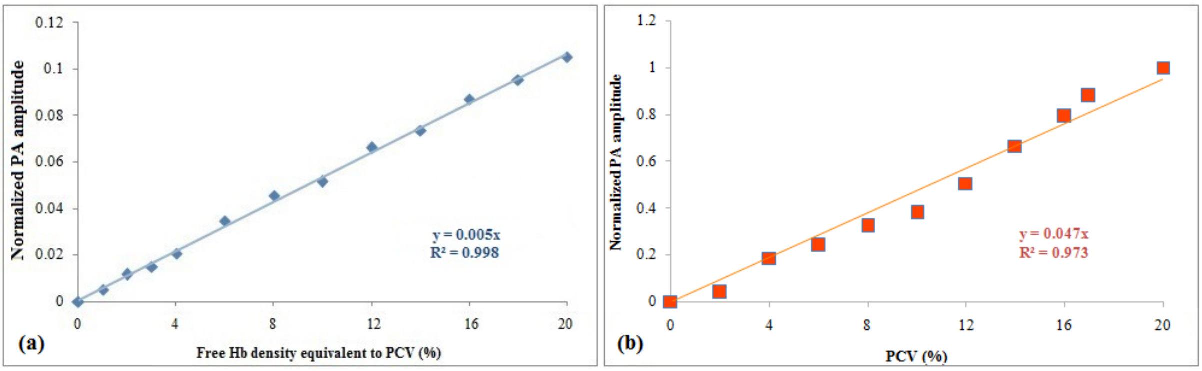

Figure S1: Variation of normalised PA amplitude with respect to (a) increasing Hb density equivalent to PCV 0% to 20% (0 for 0% is added as ideal point) and (b) increasing PCV 0% to 20% (0 for 0% is added as ideal point). An experiment has been performed to check if there is any nonlinear behaviour in these two plots which may indicate the reason behind the nonlinearity at the highly lysed end of Fig. 3. The high R^2 values of the individual linear regression suggest the PA amplitude varies linearly in both the cases. So, in Fig. 3, the nonlinearity develops may be due to the complex optical absorption of the RBCs suspension in free Hb media.

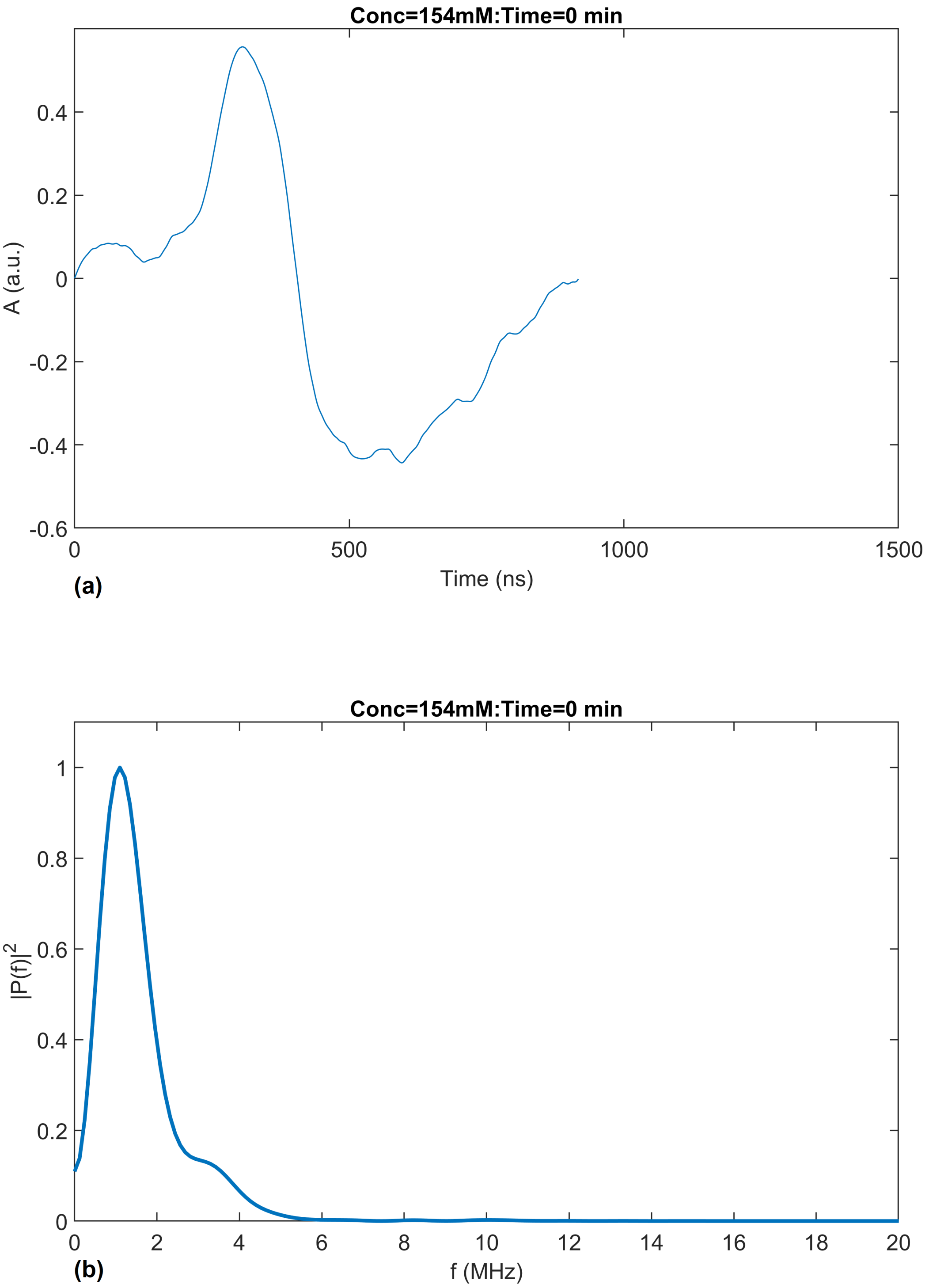

Figure S2: (a) PA Waveform of 154 mM sample, at t=0 min, self normalised, (b) power spectrum of the same, self normalised, showing peak frequency around 1.099 MHz and bandwidth around 1.366 MHz.

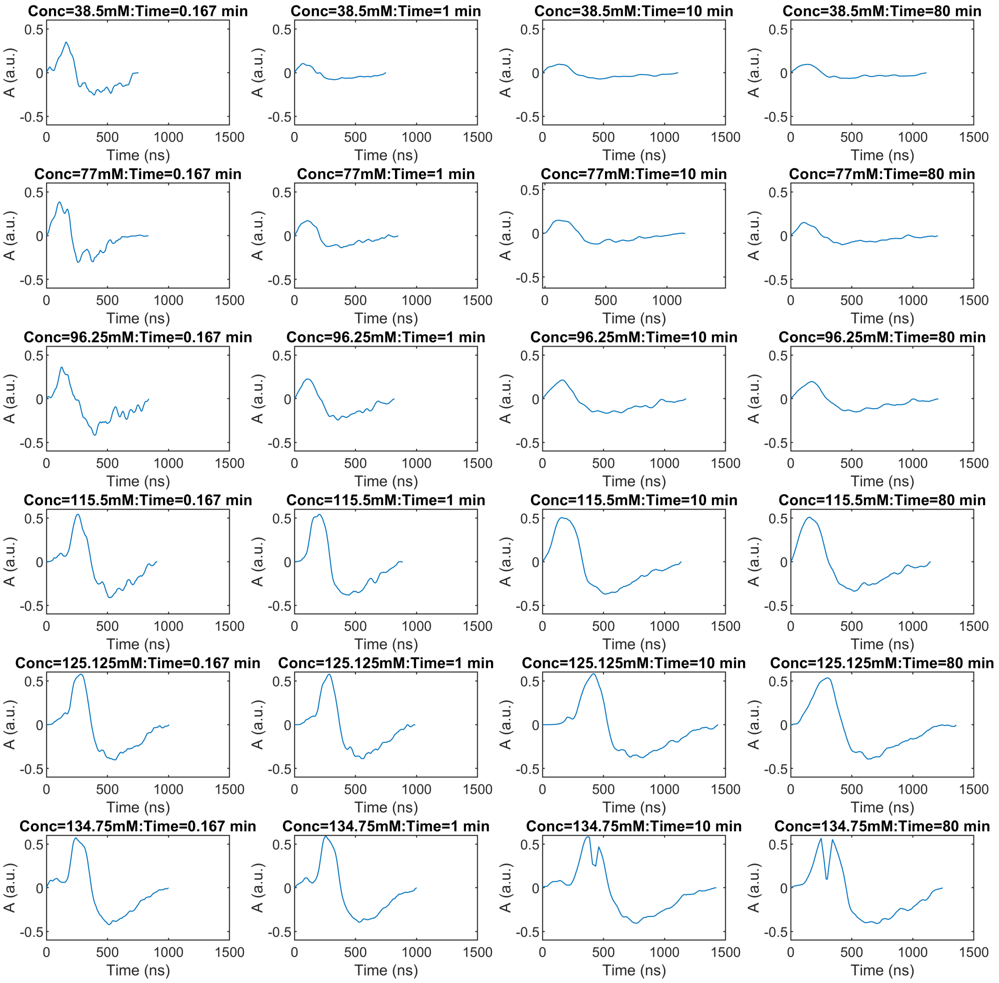

Figure S3: Exemplary waveforms drawn in the six rows are of the six hypotonic salt concentrations discussed in the manuscript, with osmolarity increasing downwards. The columns are of four time points as follows: 0.167 min, 1 min, 10 min and 80 min, chosen in a way to cover the total experiment period as well as represent the three regions of the amplitude decay namely rapid phase, transition phase and plateau.

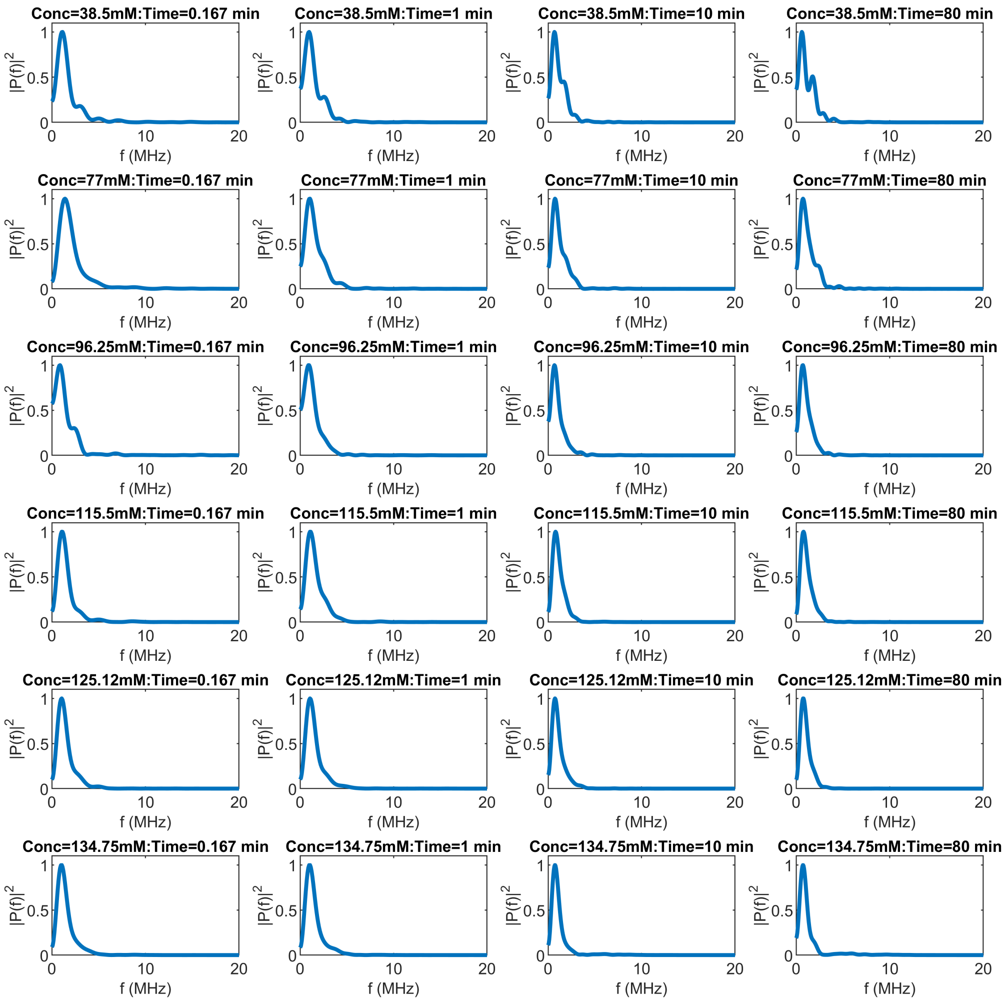

Figure S4: Exemplary power spectrums (self normalised) drawn in the six rows are of the six hypotonic salt concentrations discussed in the manuscript, with osmolarity increasing downwards. The columns are of four time points as follows: 0.167 min, 1 min, 10 min and 80 min, chosen in a way to cover the total experiment period as well as represent the three regions of the amplitude decay namely rapid phase, transition phase and plateau.

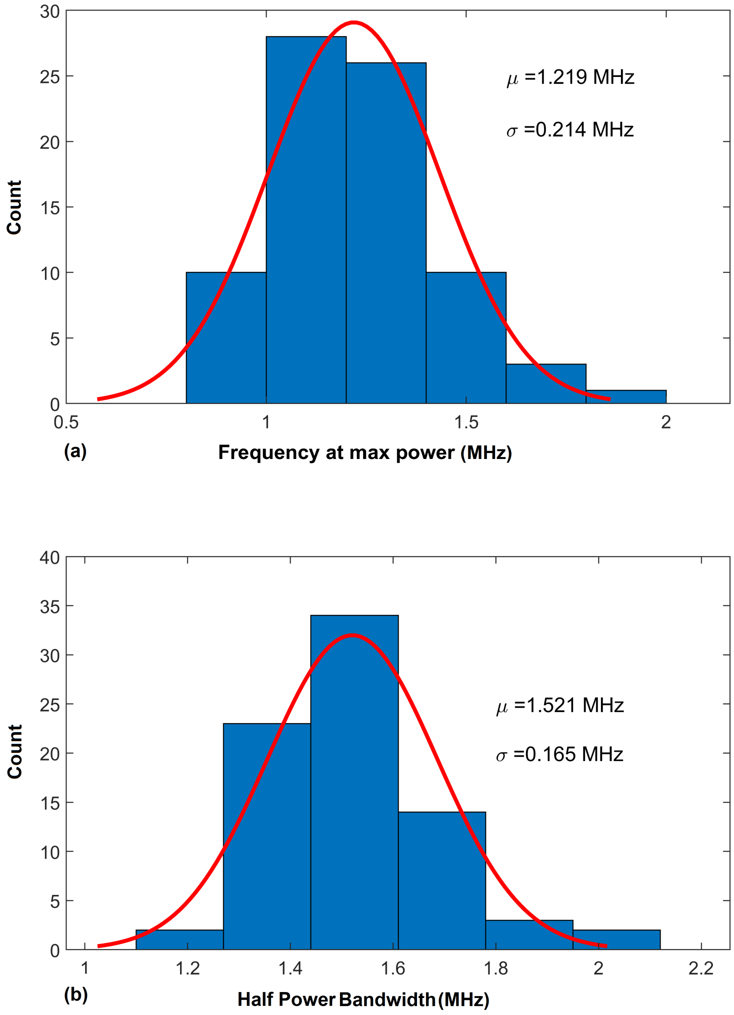

Figure S5: Histograms of (a) frequency at maximum power and (b) half power bandwidth, over 78 power spectrums of waveforms at 78 lysis points (13 waveforms for each of 6 hypotonic media). The 20% PCV, used in this experimentation, is not a sparse suspension to ignore the in-phase as well as almost in-phase addition of acoustic emission of each RBCs taking place inside the illuminated volume. PA emission by each RBC is approximately proportional to the temporal derivative of the optical pulse for long optical pulse excitation (for example, a Gaussian pulse of FWHM = 110 ns) [S1-S4], the resultant PA wave would be peaking around lower acoustic frequency (e.g. < 10 MHz). The present study has used a heavily damped, untuned (6 dB bandwidth is about 100% of the centre frequency) transducer (centre frequency: 10 MHz). The PA signals from all the hemolysing samples are found to appear at lower frequency (within 0.87 to 2 MHz). The datapoints are fitted by normal distribution; relatively small variation in the bandwidth (σ = 0.165 MHz, μ = 1.521 MHz) as well as peak frequency (σ = 0.214 MHz, μ = 1.219 MHz) is observed for different stages of lysis.

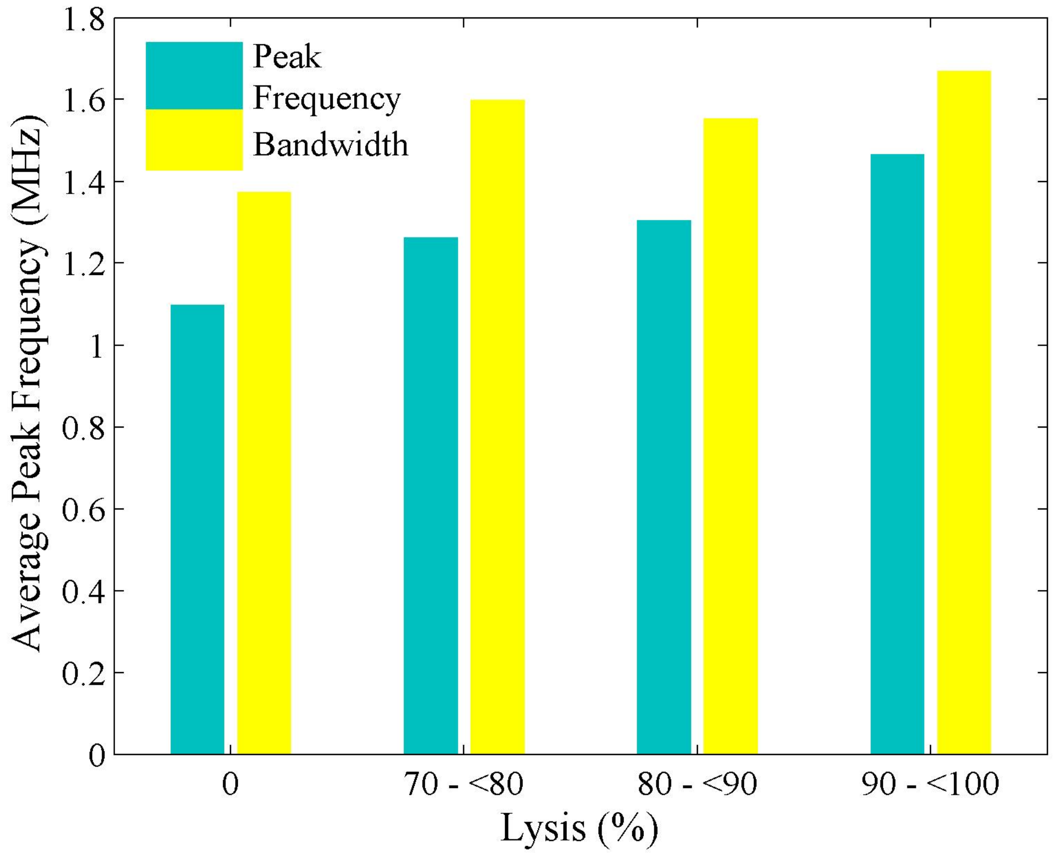

Figure S6: The average frequency at peak power (green) as well as half power bandwidth (yellow) for lysis (%) with the bins of 70-<80, 80-<90 and 90-<100 are drawn to show an increasing trend of both for this stage of lysis where the sample becomes semi-sparse to sufficiently sparse. For comparison, the frequency at peak power as well as half power bandwidth for the waveform of 154 mM, t=0min i.e. the unlysed sample is drawn at the left. The increase in frequency is observed due to the absence of in-phase or almost in-phase addition of the RBC contributions in PA response, whereas the increase in bandwidth is observed due to the increase in contribution of the free Hb background.

SUPPLEMENTARY REFERENCES:

S1. Irisawa, K., Hirasawa, T., Hirota, K.,Tsujita, K., & Ishihara, M., Influence of laser pulse width to the photoacoustic temporal waveform and the image resolution with a solid-state excitation laser, Photons Plus Ultrasound: Imaging and Sensing 8223; 82232W, 10.1117/12.907714 (2012).

S2. Calasso, I. G., Craig, W., & Diebold, G. J. Photoacoustic Point Source, *Phys. Rev. Lett.* **86**, 3550 (2001).

S3. Diebold, G. J., Sun, T., & Khan, M. I., Photoacoustic Monopole Radiation in One, Two and Three dimensions, *Phys. Rev. Lett.* **67**, 3384 (1991).

S4. Wang, W., Xing, Da., Zeng, Y. and Chen, Q., Photoacoustic imaging with deconvolution algorithm, *Phys. Med. Biol.* **49**, 3117 (2004).
